# The role of the physiotherapist in concussion

**DOI:** 10.4102/sajp.v80i1.2013

**Published:** 2024-04-30

**Authors:** Megyn K. Robertson, James McLoughlin

**Affiliations:** 1 South African Society of Physiotherapy, Johannesburg, South Africa; 2 College of Nursing and Health Sciences, Faculty of Physiotherapy, Flinders University, Adelaide, Australia

**Keywords:** concussion, physiotherapy, vestibular, oculomotor, cervicogenic, autonomic, mental health, intradisciplinary

## Abstract

In the last decade, concussion research has exploded in multiple fields of scientific research. This has helped to clarify what causes, influences, and perpetuates human concussion, and displays the integral role physiotherapists play in concussion management. In this article we discuss the latest research relevant to the key role of physiotherapy in concussion management. A narrative review of the literature on concussion was conducted. The current review analyses how concussion has influenced physiotherapy in several categories: assessment, treatment, management, research rigour and building the profile of the profession. Scientific concussion research has largely converged in support of the role of physiotherapists utilising specific components including: (1) autonomic, (2) cervicogenic, (3) vestibulo-ocular and (4) psychological approaches to management. Latest research supports the critical role of physiotherapy in concussion care in the assessment, management, and prevention of concussion with scope for further interdisciplinary collaborations.

**Clinical implications:**

Concussion is complex. A basic mental health, Vestibular Ocular Motor Screening (VOMS) and four key components relating to concussion management (autonomic, cervicogenic, vestibular oculomotor, and psychological approaches to management) should be included in the undergraduate and postgraduate curriculum. This will aid clinical physiotherapists to support their patients. A call to advance more intradisciplinary physiotherapy teamwork should be encouraged as valuable knowledge sharing is potentially lost within the framework of ‘specialisation’. If needed, the skills of a greater interdisciplinary team are imperative to facilitate patient management and recovery from this multi-faceted injury.

## A little background into concussion

Concussion is a traumatic brain injury that affects the most complex organ of our body and is an increasingly prominent public health issue (Kenzie et al. 2017). The diagnostic label ‘concussion’ may be used interchangeably with mild traumatic brain injury (mTBI) when neuroimaging is normal or not clinically indicated (Silverberg et al. 2023). A concussion is simply a violent shaking of the brain from either direct or indirect movement of the head. This causes linear and rotational stresses to be imparted to the brain and its essential constituents (cells, fibre tracts, and vasculature) that cause symptoms and signs to occur. Frequent causes of concussions are motor vehicle accidents, assault or being struck by an object, blast injuries, falls and participation in recreational sports. Much of the current research surrounding concussions stems from data on sports-related concussions (SRC). Sports-related concussions are common, but only account for 20% – 25% of all 3.7 million patients who present to casualty with a head injury in the United States annually, despite high participation rates (Harmon et al. 2013, cited in Hallock et al. 2023). Although the true incidence is not known, it has been estimated that about 33 million children worldwide sustain concussions annually (Davis et al. 2017).

Diverse concussion symptoms as well as the various factors that interact dynamically on an individual’s recovery trajectory, have resulted in many hurdles in research and clinical practice. One such hurdle is that there has been no uniformly agreed upon ‘gold standard’ definition of concussion, even though concussion research dates back to the late 19th century. Cantu (2019) writes an enlightening article on the history of concussion, revealing that the dangers of concussion were already expressed as far back as the 1870s and that in the 1970s, medical authorities had a duty to convince sporting bodies that the effects of repeated concussions were cumulative. Most definitions of concussion prior to the 1970s involved a loss of consciousness (LOC) or amnesia; however, there are numerous examples that even prior to 1924, doctors knew concussions could occur without LOC (Cantu 2019). In 2001, the first Concussion Consensus Statement defined SRC as ‘a transient change in mental status that may or may not involve LOC or amnesia’ (Aubry et al. 2002).

A second hurdle to concussion research is the lack of a benchmark diagnostic test for concussion diagnosis, or concussion resolution. For now, concussion remains a clinical diagnosis based on subjective symptom report and a selection of supportive clinical tests, such as a combination of the Vestibular Ocular Motor Screening (VOMS), Balance Error Scoring System (BESS), Post Concussion Symptoms Scale (PCSS), Standardised Assessment of Concussion (SAC), Patient Health Questionnaire (PHQ9) and Generalised Anxiety Disorder Scale (GAD7). It is therefore incumbent on the skills of the health professional assessing the patient to make a concussion diagnosis. There is emerging evidence that salivary micro-ribonucleic acids (miRNAs) may serve as biomarkers of concussion (Dadas et al. 2018; Mavroudis et al. 2023). The refinement of this approach could eventually lead to a non-invasive, sideline adjunct test for SRC (Hicks et al. 2023). Currently, it is reported that at least 50% of concussions remain undiagnosed (Harmon et al. 2013 cited in Hallock et al. 2023).

Medical clearance for return to play (RTP) is determined by symptom resolution following the completion of a graded exercise protocol (McCrory et al. 2017; Patricios et al. 2023). However, standard clinical assessments only indirectly reflect the underlying injury. There is concern that biological recovery lasts beyond symptom resolution, with long-term physical and mental health consequences that may be further exacerbated by multiple concussions (Guskiewicz et al. 2005; 2007). It is an ongoing challenge to characterise concussion pathophysiology and to determine whether recovery processes continue beyond medical clearance to RTP (Churchill et al. 2021). In light of this, we can therefore only clinically deduce when an individual has fully recovered from their concussion, and is safe to resume school, work or contact sport. The science of studying concussion is at an early stage. Therefore, decisions regarding concussion management and return to play lie largely in the realm of clinical judgement and must be made on an individual basis (McCrory et al. 2017). This creates uncertainty about the contributions of many aspects of brain health that may impact on persistent symptoms, permanent brain injury and long-term disease linked to brain trauma, such as chronic traumatic encephalopathy (CTE) (Wilson et al. 2017).

## Our understanding of concussion today

A concussion is a form of mTBI, which results in a functional disturbance as opposed to a structural injury of cerebral tissue. Diagnostic criteria for mTBI now consider mechanism of injury, clinical signs, symptoms, and neuroimaging. Rotational acceleration–deceleration injuries are believed to be the primary injury mechanism for brain strain. Given its physical properties, brain tissue deforms more easily when exposed to shear forces (Meaney & Smith 2011). This biomechanical injury results in a complex cascade of neurochemical and neurometabolic events, namely ionic flux, proliferating glutamate release, activating immune receptors, and changing inflammatory markers, resulting in a pervasive depression-like state in the brain (Giza & Hovda 2014). In an effort to restore ionic and cellular homeostasis, hyperglycolysis occurs, with relative depletion of intracellular energy reserves resulting in a metabolic crisis. This energy crisis in conjunction with reduced cerebral blood flow, creates a mismatch between energy supply and demand (Giza & Hovda 2014).

Scientific research on concussion has largely converged in support of seven key concepts that have fundamentally shifted physiotherapists’ understanding and treatment of concussion (Box 1). Common symptoms of concussion include headaches, dizziness, anxiety, depression, blurred vision, poor concentration, memory disturbance, noise and/or light sensitivity, and problems with sleep (McCrory et al. 2017; Patricios et al. 2023; Silverberg et al. 2023). These clinical signs and symptoms cannot be explained by drug, alcohol, or medication use, other injuries (such as cervical injuries, peripheral vestibular dysfunction, etc.) or other comorbidities (e.g. psychological factors or coexisting medical conditions) (Silverberg et al. 2023). Resolution of the clinical and cognitive features typically follows a sequential course. However, in approximately one-third of cases, symptoms may be prolonged (Van der Vlegel et al. 2021). The best concussion assessments today are those that involve tests for multiple brain domains and include autonomic, neurological, vestibulo-ocular, neurocognitive, psychological, and cervical aspects (Feddermann-Demont et al. 2017; Mucha & Trbovich 2019).

BOX 1 Key concepts that have shifted physiotherapists’ understanding of concussion.

1. Concussion is a metabolic injury. To date there is no single commercially available test to diagnose concussion, and therefore concussion remains a clinical diagnosis (Deakin, Suckling & Hutchinson 2021; Mann, Tator & Carson 2017; Silverberg et al. 2023).
2. 70% – 80% of patients with concussion make a symptomatic recovery within 10–14 days (adults)/15–30 days (children and adolescents) (McCrory et al. 2017). Physiotherapists have a responsibility to highlight that 20% – 30% who remain symptomatic for a prolonged period with significant functional limitations, to prevent chronic sequelae.
3. Females are more susceptible to concussion (Kerr et al. 2019; Schallmo et al. 2017), have more severe concussions, have more delayed recoveries and are at a higher risk of persistent symptoms (Gupte et al. 2019).
4. The number, duration, and severity of total post-concussion symptoms are important in determining concussion severity. The combination of symptoms is more important than the single symptom of amnesia or loss of consciousness. In almost 90% of cases, there is no loss of consciousness (Iverson et al. 2017; Scopaz & Hatzenbuehler 2013).
5. Accumulative forces in the form of sub-concussive blows increase the risk of chronic traumatic encephalopathy (CTE), rather than number of concussions sustained (Johnson et al. 2014).
6. Additional risk factors for prolonged recovery include younger age, a pre-existing history of mental health issues, learning disabilities, migraine, and vestibulo ocular dysfunction (Kontos et al. 2013; Lau et al. 2011; Morgan et al. 2015; Mucha & Trbovich 2019; Pearce et al. 2015; Van der Naalt et al. 2017; Zemek & Yeates 2017).
7. Current concussion treatment protocol recommends 24–48 h of complete rest followed by early physiotherapy interventions, including vestibular and neuromusculoskeletal therapy, with concurrent sub-symptom threshold cardiovascular exercise (Schneider et al. 2017).

### Autonomic nervous system

The mechanism of action between concussion and autonomic nervous system (ANS) dysfunction is yet to be fully elucidated. However, dysfunction of the ANS is suggested as a significant impairment to consider when assessing an individual with concussion, and is a factor that can contribute to persistent post concussive complaints (Leddy et al. 2016; Pertab et al. 2018). For many rehabilitation professionals, training regarding the workings of the ANS tends to be focused on the fight-flight and rest-digest aspects of the sympathetic nervous systems (SNS) and parasympathetic nervous systems (PNS) (Pertab et al. 2018). Autonomic nervous system dysregulation has the potential to impact baroreflex efficiency, cerebral perfusion and metabolic demands of the brain following concussion, as its capacity to respond to changing conditions is compromised (Pertab et al. 2018). The ANS plays a role in regulating blood pressure, body temperature, glucose metabolism, and many other physical processes (Gordan, Gwathmey & Xie 2015; Pertab et al. 2018). Abnormal responses to blood pressure, position change and exercise create symptoms such as dizziness, light-headedness and headaches, which may make it difficult to distinguish between diagnoses like postural orthostatic tachycardia syndrome (POTS) or physiologic post-concussion disorders (Miranda et al. 2018).

A common yet debilitating symptom of acute and persistent concussion is exercise intolerance. These patients are unable to exercise to their age-expected maximal heart rate without symptom exacerbation (Janssen, Pope & Rando 2022). This mechanism describes a neuro-autonomic cardiovascular dysfunction whereby the ANS causes altered signals to go to the heart. Graded exertional testing, such as the Buffalo Concussion Treadmill Test (BCTT) (Haider et al. 2019; Janssen et al. 2022; Mucha et al. 2019) has emerged as a way of studying the connection between the ANS and concussion through measuring blood pressure responses and heart rate (Esterov & Greenwald 2017; Leddy et al. 2007). Heart rate variability (HRV) disturbances post-concussion in athletes have been found to persist beyond symptom resolution and return to play protocols as well. Subthreshold aerobic exercise appears to lower symptom scores in concussed individuals, but not time to recovery (Kozlowski et al. 2013; Reid, Farbenblum & McLeod 2022). Individually tailored multimodal interventions have a worthwhile effect in providing faster return to sport and clinical improvement, specifically in those with persistent symptoms (Miranda et al. 2018; Reid et al. 2022).

Peripheral ANS structures vulnerable to forces involved in concussion include the cervical sympathetic chain (SNS), and the vagus nerve and its branches (PNS), which pass through the cervical spine region. Damage to cervical spinal structures in injuries such as whiplash and cervical strain are known to correlate with ANS anomalies including dysfunction in the cervical sympathetic chain in some patients (Adeboye, Emerton & Hughes 2000; Sterling 2011). There is marked overlap between the symptoms of concussion and cervical strain, or whiplash injuries, and a growing body of research supports increased clinical attention to neck pathology in patients with concussion (Cheever et al. 2016; Morgan et al. 2015).

### Cervicogenic system

The cervical spine is a functional, multi-planar and sensorimotor organ (Worsfold 2020). Cervical injuries and concussion can share similar mechanisms and nearly identical symptoms or causes, making it difficult to differentiate between patients with a concussion and patients with cervical injuries. Reduced range of motion, proprioceptive errors and oculomotor signs are linked with cervical mechanical dysfunction; these are predictors of persistent symptoms because of concussion and cervical injury (Cheever et al. 2016; Morgan et al. 2015). Injuries to the cervical spine are a potential source of headache, dizziness, balance disturbances, neck pain and stiffness. The cervicogenic system may therefore contribute to persistent post-concussion symptoms. Mohai et al. (2022) identified that only 13% of standardised concussion evaluation tools included a cervical spine evaluation component. This indicates that potential cervical spine injury is either not considered, or is considered separately from concussion evaluation tools. It is pertinent to assess the cervical spine and cranial nerves to identify any cervical spine or vascular intracerebral injuries (Kennedy et al. 2017). Other musculoskeletal examination elements to consider include the temporomandibular joint and thoracic spine.

After a head and neck patho-mechanical event, specific clinical tests should be incorporated in the diagnostic portion of a concussion screening. These tests identify any deficits in sensorimotor integration, cervical function and cervicogenic symptoms. The reproduction of symptoms, loss of range of motion or loss of motor-control accuracy during testing can then be attributed to cervical spine involvement. Incorrect afferent output from the cervical spine (e.g. from muscle spindles and/or mechanoreceptors) can lead to mismatches between the cervico-ocular and vestibulo-ocular reflexes, manifesting as unsteadiness, dizziness, and vision-related disturbance. Thus, cervical proprioception, eye movement control, postural stability and movement velocity and trajectory of the head are impaired in neck pain (Treleaven 2008; Treleaven et al. 2008). The five identified cervicogenic tests recommended include: the cervical joint position error test (JPET), the smooth pursuit neck torsion test (SPNTT), the head neck differentiation test (HNDT), the cervical flexion rotation test (CFRT), and motor control assessment of deep cervical flexors and extensors (Treleaven 2008, Treleaven, Jull & LowChoy 2006, Treleaven, Jull & Sterling 2003).

### Vestibular and oculomotor system

Vertigo, dizziness, and balance dysfunction persist in 10% – 30% of concussion cases (Bowman et al. 2024; Murray et al. 2017). Patient complaints can include difficulty focusing, elevated motion sensitivity, and imbalance in visually stimulating environments such as the mall or supermarket or when walking on uneven surfaces. Post-traumatic benign positional paroxysmal vertigo (BPPV) is not uncommon after concussion (Wang et al. 2021). The BPPV that results from a concussion may be more difficult to treat than idiopathic BPPV. As a result of the forceful nature of the injury, there may often be more than one canal involved, more than one treatment may be required, and the frequency of recurrence is greater. Repositioning manoeuvres may need to be performed more than once to effectively clear the canal (Ahn et al. 2011).

The oculomotor system, alongside the vestibular system, produce a variety of eye movements responsible for visual fixation of an image on the fovea. The vestibulo-ocular and vestibulo-spinal tracts are susceptible to the shearing forces in head injury and can be linked to many symptoms and activity limitations in concussion. The most commonly affected visual fixation systems in concussion include smooth pursuit, saccades, near-point convergence, the vestibulo-ocular reflex (VOR), and accommodation (Armstrong 2018; Kontos et al. 2017; McDonald, Holdsworth & Danesh-Meyer 2022). Deficits in these systems may create symptoms such as headache, nausea, blurred or double vision, dizziness, difficulty reading or scrolling on a computer, light sensitivity, and eyestrain, all of which impair work or school performance. The VOMS tool is a sensitive tool to support diagnosis of concussion, has demonstrated high internal consistency (Mucha et al. 2014), and correlated significantly with the PCSS (Ferris et al. 2022). It was developed to identify and measure the subjective symptoms of headache, dizziness, nausea, and fogginess related to vestibular and oculomotor impairments following concussion. The assessment focuses on symptom provocation of the most common oculomotor deficits including smooth pursuit, saccades, near point convergence, VOR, and visual motion sensitivity.

Vestibular rehabilitation therapy (VRT) is an exercise-based intervention designed to address specific vestibular and oculomotor deficits identified in the examination (Babula et al. 2023; Lacour & Bernard-Demanze 2015). Vestibular rehabilitation therapy can be grouped into exercises that focus on oculomotor function (particularly gaze stability and balance retraining) and canal repositioning manoeuvres. Gaze stabilisation exercises in concussion are a combination of adaptation exercises designed to drive recovery and minimise symptom provocation through repeated exposure of the stimulus. Each exercise is based on the goal of maintaining clear visual focus on a target while the target, the head, or both are moving. Gait and balance retraining through sensorimotor integration, and habituation exercises for motion sensitivity are included. Optimal prescription and efficacy of VRT in patients with concussion is still a work in progress (Murray et al. 2017). However, there is good evidence to support that earlier vestibular intervention for concussion has a direct relationship on earlier recovery and return to sport (Ferry et al. 2023). There is evolving evidence to show that individually tailored multimodal interventions including cervical, vestibular and/or oculomotor therapies provide a faster return to sport and clinical improvement, especially in those with persistent symptoms (Galeno et al. 2022; Reid et al. 2022; Reneker et al. 2017; Schneider et al. 2014) and that sub-symptom aerobic exercise lowers symptom scores (Reid et al. 2022), but we need larger samples to validate this. Future research projects may also need to consider the role of physiotherapy in combination with virtual reality and other multimodal domains (McLoughlin 2023).

### Psychological approaches to pain management

Post-traumatic headache (PTH) is a common secondary headache that commences within 7 days of a concussion (Zemek et al. 2016). The PTH includes a broad range of phenotypes that can include not only migraine-like headaches, tension type headaches and trigeminal neuralgias but can also be complicated by medication overuse. The pathophysiology of PTH is multifactorial. Acute PTH may be attributed to increased peripheral pain sensitisation with impaired pain inhibiting pathways. Chronic or persistent PTH may be because of a chronic inflammatory response and peripheral as well as central sensitisation (Blumenfeld, McVige & Knievel 2022). It is therefore prudent to consider differential diagnoses such as somatisation, chronic fatigue, chronic pain, or some combination of these conditions. Fear-avoidance beliefs and catastrophising may play an active part in the transition from acute to chronic pain in the concussion spectrum (Green et al. 2022; Sigurdardottir et al. 2013). Physiotherapists’ understanding of pain behaviours, screening, and early intervention relevant to other clinical areas such as chronic low back pain are likely to be very valuable (Gatchel et al. 2018).

There is an established link between concussion and poor mental health outcomes (Auclair-Pilote et al. 2021) in both the paediatric and adult populations. The definition of mental health according to the World Health Organization (WHO) is a state of mental well-being, that enables individuals to cope with difficulties in life, understanding their abilities, and working towards the betterment of themselves as well as for the community (World Health Organization 2022). Persistent post-concussion symptoms can make daily functioning significantly harder. Adolescents specifically, are at an increased risk of higher depressive symptoms and self-injurious behaviours (Yang et al. 2019 cited in Sheldrake et al. 2022). There are various hypotheses to potentially explain this causal link between concussion and poor mental health. These include: concussion is a type of brain depression that directly results in mood dysregulation and symptoms like depression can develop after head injury (McCrory et al. 2017); reduced activities or participation associated with the post-concussive state have psychosocial implications that could lead to poor mental health outcomes (Sheldrake et al. 2022); and pre-injury family and personal psychiatric history can increase susceptibility to future mental health conditions post-injury (Fralick et al. 2019; Ledoux et al. 2022; Shahrestani et al. 2022).

Sleep dysregulation is also associated with persistent concussion symptoms such as impaired executive function, reduced working memory and information processing speed (McCrea, Nelson & Guskiewicz 2017). Poor sleep quality is known to increase negative affect and emotional reactivity and can be a predictor of emotional symptoms in adults and children (Theadom et al. 2018). Sleep must be considered for its role in mental health. Physiotherapists have a responsibility to identify this patient population with mental health difficulties post-concussion and refer to the relevant healthcare professionals for support.

## Concussion treatment

An awareness of all the relationships at play increases our understanding of the physical impact of concussion. It partially explains the overlap of concussion symptoms with other medical conditions such as migraine, depression, anxiety, diplopia, vasovagal attacks, motion sickness, sleep disruption and chronic fatigue, to name a few. Damage to the maturing brain of a young athlete can be catastrophic, that is, almost all reported cases of death because of second impact syndrome (SIS) are in young athletes (Bey & Ostick 2009). Athletes under 18 years of age should be managed more conservatively, using stricter RTP guidelines than those used in the more mature athlete (Davis et al. 2017; DeMatteo et al. 2020; Elbin et al. 2016; Podolak et al. 2021; Terwilliger et al. 2016).

Individuals with concussion rarely fit into only one pathway. Early physiotherapy interventions that are individualised, patient-centred and incorporate autonomic, vestibular and cervicogenic systems into treatments are effective in reducing persistent symptoms, with a moderate effect for subthreshold aerobic exercise, and promising effect of multimodal cervical and vestibular physical rehabilitation (Quatman-Yates et al. 2020; Reid et al. 2022). Schneider et al. (2014) showed that participants who underwent combined vestibular and cervical spine rehabilitation programmes were 3.91 times more likely to return to participation in less than 8 weeks than patients who did not partake in therapy. Patients who incorporate earlier physical activity and receive active physiotherapy interventions post-concussion have reduced symptoms and optimise their recovery time (Art et al. 2023; Quatman-Yates et al. 2020; Schneider et al. 2017, 2023).

Physiotherapists are pivotal in educating the child, caregiver, and adult regarding the process of returning to activity. Impairments and activity limitations that have the greatest impact should be addressed first in the plan of care (Quatman-Yates et al. 2020). Return to learn (RTL) and return to play (RTP) protocols can be run in parallel. Patients progress through each stage for a minimum of 24 h, as long as their symptoms are not exacerbated, until they are back to full workload and contact sport. A constructive, shared treatment plan alleviates stress and provides huge reassurance around what to expect in the days or weeks to come. This presents great opportunities for further research and has the potential to powerfully inform treatment decisions (Pertab et al. 2018). Running physiotherapy interventions in parallel with current RTP guidelines is a simple example of a next step in implementing best practice (Teare-Ketter, Ebert & Todd 2023).

## The future of concussion

Clinicians face the difficult task of interpreting which symptoms are directly related to concussion, and which are related to other conditions. A headache is a simple example of a very common symptom of concussion. It may be the result of a concussive injury or referred pain from a neck injury. A headache can be a symptom of hypertension, or cranial nerve fallout, or simply a symptom of dehydration post-exercise. A headache can be directly related to medication overuse or a migraine or simply wearing a helmet. Differentiating the symptoms of concussion and cervical injury, for example, is a vital part of concussion screening to ensure appropriate diagnosis, management, and treatment. A broad foundation of assessment tools is necessary to identify and treat a plethora of domains (e.g. balance, headache, neck pain, dizziness, vertigo). As frontline practitioners, physiotherapists are fully equipped to understand each patient’s clinical presentation, especially when considering recovery and quality of life post-concussion. Physiotherapy clinicians focus not only on the evidence around sensitivity and specificity of diagnostic concussion tests, but just as importantly, they focus on the best evidence around assessment and targeted treatment of the relevant domains that may be impaired following a concussion. While mental health is not technically in the physiotherapy realm, physiotherapists play a critical role in supporting patients, advocating for better coping skills, managing expectations and goal setting. Psychological affect needs to be considered as inclusive to recovery, especially school children who are anxious about writing exams, and those patients who demonstrate fear avoidance behaviours, or any factors that precipitate neural inflammation and chronic pain. We need to utilise this information in our biopsychosocial approach, uncovering root causes of various signs and symptoms of concussion. The use of language, like ‘persistent symptoms’ rather than ‘post-concussion syndrome’ is a step in the right direction. We need to be responsible when referring to screening for mental health, considering that mental health influences outcomes of interventions in vestibular, cervical rehabilitation and exercise (Moore, Stark & D’Angelo 2023).

Clinical physiotherapists reporting on their outcomes are in the unique position of bridging the gap between research and clinical practice, to reduce incidence and severity of concussion, improve outcomes that speed up recovery and reduce persistent symptom impact on quality of life. A description and rationale for dosage is essential, for example, the number of sessions, intensity, and duration of the intervention whether it be education, exercise or manipulative therapy (Jull & Moore 2021). Currently, reporting of the dose and dosage of gaze stabilisation interventions is infrequent, impairing the ability to translate current evidence into clinical care. Improved reporting and use of outcome measures are vital to establish optimal intervention parameters for those with gaze stability impairments (Cole, Goodman & Volland 2022).

With the advent of speciality groups in physiotherapy in 1974 (Lamb et al. 2003; Lonnemann, Olson & Brismée 2017), physiotherapists now work in separate bodies (e.g. musculoskeletal, neurology). Speciality physiotherapists have a wealth of knowledge to contribute within their respective specialities. However, sharing of knowledge or experiences may not be optimal with other speciality groups. Perhaps, concussion is our opportunity to work in speciality intradisciplinary teams, an opportunity for physiotherapists to be more innovative and consider more combined therapies and technologies that enhance both assessment and rehabilitation within our own profession (McLoughlin 2023). An exchange of ideas and cooperation between authors of sport and non-sport concussion will enrich the overall knowledge and treatment of concussion (Sojka 2011). The Sports Concussion Assessment Tool (SCAT6) is a standardised tool for evaluating sports-related concussions and is intended for use in the acute phase, ideally within 72 h of injury (Echemendia et al. 2023). Sports-related concussions contribute approximately 25% of all concussions (Harman et al, 2013 cited in Hallock et al. 2023; Theodom et al. 2020) when the concussion cohort is inclusive of falls, motor vehicle accidents, blast injuries and intimate partner violence. Very few studies utilise the SCAT6 tool in non-athlete populations (Toman et al. 2022). Perhaps inclusion of the SCAT6 in the whole concussion population will limit the number of patients with missed concussion diagnoses, and prevent that 20% – 30% of the concussion population with persistent concussive symptoms (Van der Vlegel et al. 2021; Zemek & Yeates 2017).

Clinicians are encouraged to work more closely with researchers to answer important questions and drive excellence. There is a real need for studies of concussions to understand the complexities arising from the unique nature of the developing brain, the female brain and the disabled brain (Kuemmerle & Meehan, 2019). Evidence based on practice is the gold standard in patient care, yet it takes more than a decade to implant research results into clinical practice (Kristensen et al., 2016). An imprecise classification system of concussion, a dearth of high-quality clinical trials, and a lack of shared, evidence-based working model of concussion pathophysiology and recovery within the medical community hinder our effectiveness in concussion care. A shared conceptual framework for concussion is needed to facilitate interdisciplinary communication and understanding of concussion (Kenzie et al., 2017).

## Conclusion

Our understanding of concussion and best practice continues to evolve as research advances and media attention grows. The World Confederation of Physical Therapy (WCPT) Policy Statement (2019) aptly describes the profession of physiotherapy as maximising quality of life and movement potential within the spheres of promotion, prevention, treatment, and rehabilitation. A concussion diagnosis requires a multi-faceted assessment, incorporating autonomic, neurological, vestibulo-ocular, neurocognitive, cervical and psychological aspects (Feddermann-Demont et al. 2017). It is our responsibility to provide concussion awareness education to our communities, as well as to diagnose and treat concussion-related deficits. The physiotherapy community can be the catalyst for a paradigm shift in our conceptual and practical framework of concussion management, in all its complexity.
